# *In Vivo* Positron Emission Tomography Imaging of Adenosine A_2A_ Receptors

**DOI:** 10.3389/fphar.2020.599857

**Published:** 2020-11-26

**Authors:** Meng-Juan Sun, Fang Liu, Ya-Fei Zhao, Xiao-Ai Wu

**Affiliations:** ^1^ Chengdu University of Traditional Chinese Medicine, Chengdu, China; ^2^ Acupuncture and Chronobiology Key Laboratory of Sichuan Province, Chengdu, China; ^3^ Department of Laboratory Pathology, Xijing Hospital, Fourth Military Medical University, Xian, China; ^4^ Department of Nuclear Medicine, Laboratory of Clinical Nuclear Medicine, West China Hospital, Sichuan University, Chengdu, China

**Keywords:** adenosine A2A receptors, positron emission tomography tracers, positron emission tomography imaging, neurodegenerative and psychiatric disease, autoimmune diseases, cardiovascular diseases

## Abstract

As an invasive nuclear medical imaging technology, positron emission tomography (PET) possess the possibility to imaging the distribution as well as the density of selective receptors via specific PET tracers. Inspired by PET, the development of radio-chemistry has greatly promoted the progress of innovative imaging PET tracers for adenosine receptors, in particular adenosine A2A receptors (A_2A_Rs). PET imaging of A2A receptors play import roles in the research of adenosine related disorders. Several radio-tracers for A_2A_ receptors imaging have been evaluated in human studies. This paper reviews the recent research progress of PET tracers for A2A receptors imaging, and their applications in the diagnosis and treatment of related disease, such as cardiovascular diseases, autoimmune diseases, neurodegenerative and psychiatric disease. The future development of A2A PET tracers were also discussed.

## Introduction

As an extracellular endogenous messenger, adenosine play important roles in biochemical processes, signal transduction and neurotransmission (Estrela and Abraham, 2011). In physiological and pathological conditions, it acts as a cytoprotectant and a neuromodulator in response to organ and tissue stress (Khanapur et al., 2013). It also holds the capability to reduce energy demand or increase energy supply to organs or tissues which are damaged or disturbed. It is known that cytoprotective and neuromodulatory function in the brain are mediated by four adenosine receptors (ARs), namely A_1_, A_2A_, A_2B_, and A_3_ (Jacobson and Gao, 2006; Khanapur et al., 2013). A2ARs are ubiquitously distributed in brain, heart, lungs and spleen, and A2ARs mainly facilitates neurotransmissions and other physiological functions. A_2A_Rs are involved in multiple physiological processes (Tang et al., 2019; Chen and Cunha, 2020), as well as in various pathological conditions (Illes et al., 2016; Burnstock, 2017). The dysfunction of A_2A_Rs are related to many diseases such as cardiovascular diseases, autoimmune Diseases, neurodegenerative and psychiatric disease. However, many of their functions in pathophysiological processes remain unknown, partly due to the lack of available techniques for spatial and temporal control of purinergic signaling. Positron emission tomography (PET) is a nuclear medical technology that allows *in vivo* imaging and quantification of specific targets, as well as molecular and cellular processes in the living body. For example, with specific brain-targeted radio-tracers, PET therefore enables the *in vivo* imaging of local brain function, including receptor-binding ability, cerebral blood flow, and molecular metabolism (Mishina and Ishiwata, 2014).

**FIGURE 1:**
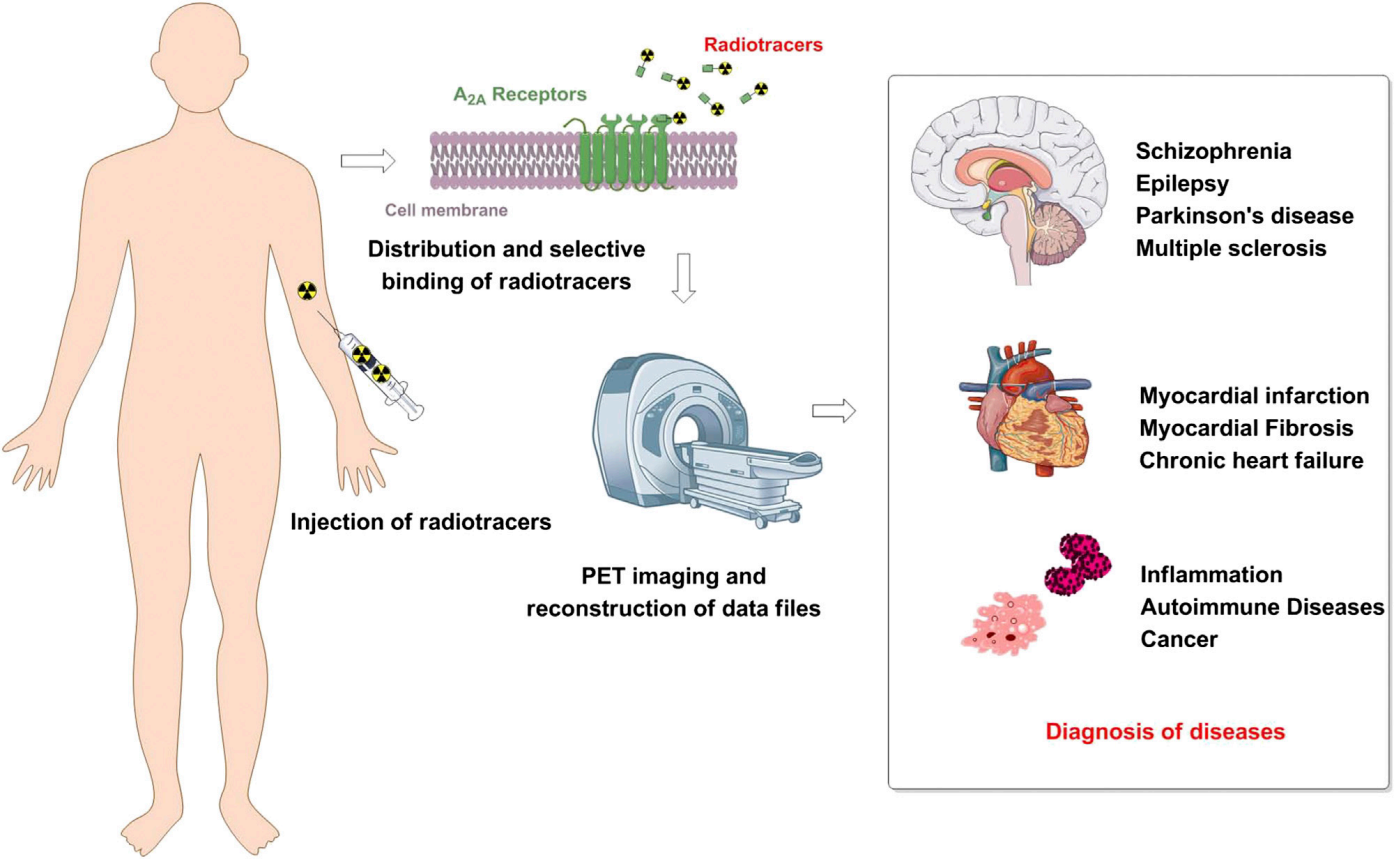
Schematic showing the diagnosis values of A_2A_R positron emission tomography (PET) imaging.

At present, PET imaging studies on adenosine receptors are mainly focused on A1 and A2A receptors, and for the diagnose of related diseases (Figure 1). In this paper, we will discuss the recent progress of lead compounds and related radio tracers for PET imaging for A_2A_Rs. In addition, this review also outlines PET imaging for adenosine A_2A_ receptors in health and diseases subjects. Furthermore, the direction of future development of A_2A_ PET tracers were also discussed.

## Development of Adenosine A_2A_ Based Positron Emission Tomography Tracers

**FIGURE 2:**
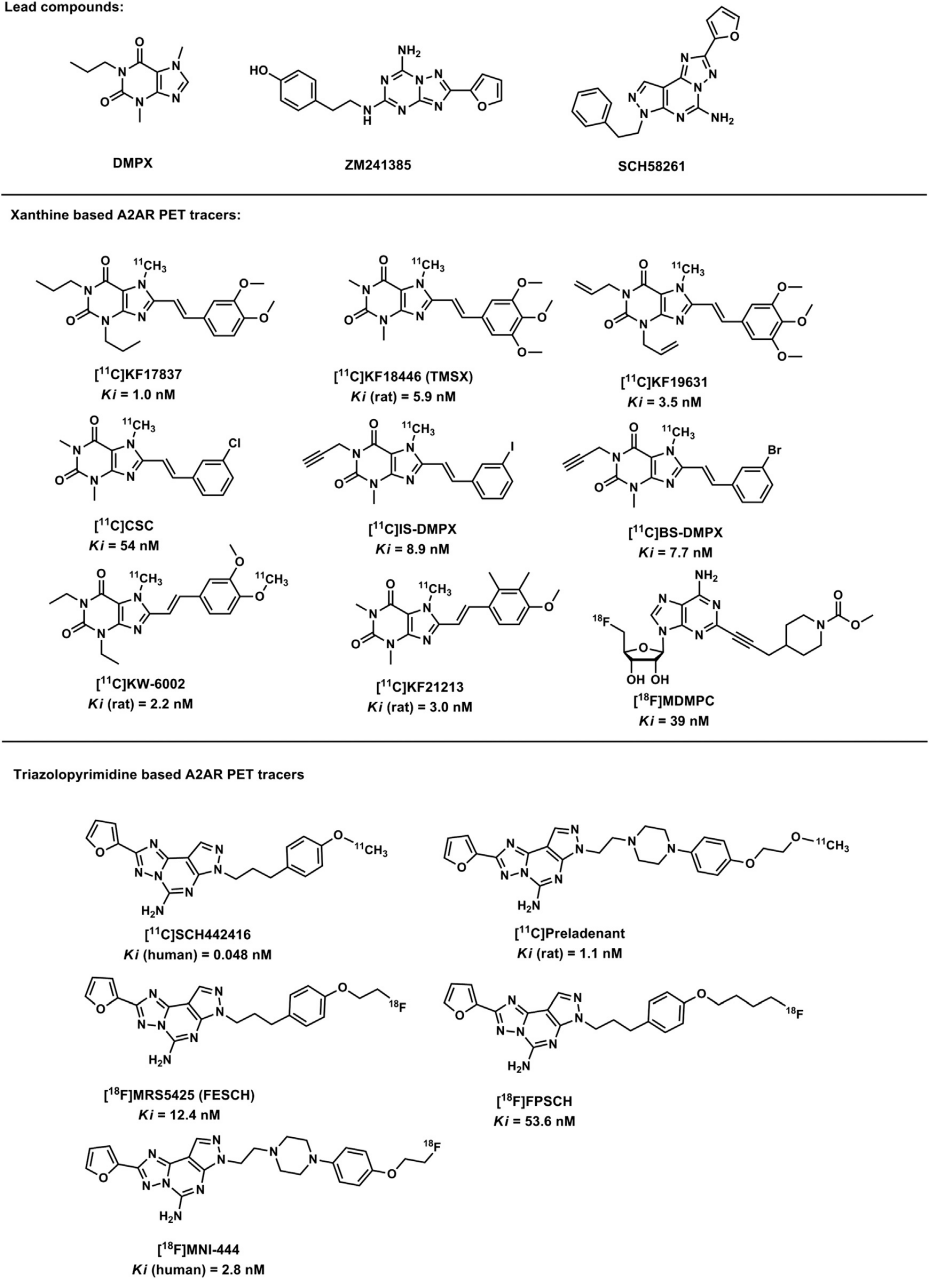
Structures of the lead compounds and the A_2A_R radiotracers.

In 1988, 3,7-dimethyl-1-propylxanthine (DMPX) was identified as an A_2A_R-targeted selective antagonists (Seale et al., 1988), several xanthine based radio-tracers were also successfully developed thereafter. In addition, shortly after the discovery and report of a novel pyrazolol-pyrimidine based compound as a potent and selective A_2A_R antagonist (Poucher et al., 1995; Baraldi et al., 1996; Zocchi et al., 1996; Baraldi et al., 1998), these compounds with a fused heterocycles were also regarded as lead compounds for A_2A_R PET tracers (Figure 2). Therefore, current PET tracers for A_2A_ receptors can be subdivided into the following two categories (Figure 2): 1. xanthine based A_2A_R PET tracers; 2. triazolopyrimidine based A_2A_R PET tracers.

### Development of Xanthine Based A_2A_R Positron Emission Tomography Tracers

With the similar chemical structure of A_2A_R endogenous ligand purine, xanthine and its derivatives showed promising properties in the A_2A_R PET imaging. Shimada et al. have identified that xanthine bearing the styryl group showed selective A_2A_R antagonistic properties (Schiffmann et al., 1991), and KF17837, a ligand with superior selectivity and potent affinity for A_2A_Rs, was optimized for the development A_2A_R PET tracers (Seale et al., 1988). At present, several PET tracers were reported, such as [11C]TMSX ([11C]KF18446) (Ishiwata et al., 2000a; Ishiwata et al., 2003b), [11C]KF19631, [11C]CSC, [11C]BS-DMPX, [11C]IS-DMPX (Ishiwata et al., 2000b), [11C]KW-6002, [11C]KF21213 (Wang et al., 2000) and [18F]MDMPC (Lowe et al., 2017), and were investigated as promising PET agents (Ishiwata et al., 1996; Stone-Elander et al., 1997; Wang et al., 2000). In addition, [11C]TMSX (formally designated as [11C]KF18446) was selected for medical applications (Ishiwata et al., 2005). After the discovery of [11C]KW-6002 (Hirani et al., 2001), its reference standard KW-6002 (with commercial name istradefylline), was developed as an anti-PD agent (Hauser et al., 2003; Bara-Jimenez, W et al., 2003). Compared with [11C]TMSX, [11C]KF21213 showed a slightly higher affinity but an improved selectivity over other ARs (Wang et al., 2000). However, [11C]KF21213 has not been evaluated in human research. However, studies also indicated that the styryl group will lead to the photoisomerization for almost all xanthine-type adenosine A_2A_R-selective ligands (Merskey, 1983; Ishiwata et al., 2003b).

### Triazolopyrimidine Based A2 Adenosine Receptor Positron Emission Tomography Tracers

Based on the findings of antagonism for A_2A_R from triazolopyrimidine based compounds such as ZM241385 (Poucher et al., 1995) and SCH58261 (Zocchi et al., 1996), another class of A_2A_R PET tracers were developed based on the novel triazolopyrimidine structure. Todde et al. prepared [11C]SCH442416 by O-methylation (Todde et al., 2000), and this radio-ligand exhibits the highest selectivity and affinity among all A_2A_ PET ligands reported as present. In addition, several nonxanthine heterocycles have also been synthesized and radiolabeled for A_2A_R PET imaging, including [18F]FESCH ([18F]MRS5425) (Bhattacharjee et al., 2011), [18F]FPSCH (Fastbom et al., 1998; Khanapur et al., 2017), [18F]MNI-444 (Barret et al., 2014, Barret et al., 2015), [18F]MDMPC (Lowe et al., 2017) and [11C]preladenant (Zhou et al., 2014). S. Khanapure et al. (Khanapur et al., 2017) reported the discovery of [18F]FESCH and [18F]FPSCH analogs and the evaluation in normal rats (Khanapur et al., 2014). Preliminary investigations of these tracers revealed a similar distribution pattern with the known expressions of A_2A_R in rat brain (Khanapur et al., 2017). Labeled with 18F, [18F]FPSCH provided more convenience in imaging protocols, as well as tracer kinetics files similar to [11C]preladenant. Compared with [18F]FESCH, [18F]FPSCH exhibited lower striatal SRTM BP_ND_ value. Dynamic PET imaging suggesting [18F]FESCH is the most favorable PET ligand for *in vivo* quantitation of A_2A_R distribution in the rodent brain.

Among all xanthine and non-xanthine based ligands, the most potent affinity for A_2A_Rs was observed in SCH442416. IS–DMPX, KF21213 and SCH442416 exhibited higher A_2A_R selectivity. The selective uptake in striatum was observed in validation studies in rodents for all radio-labeled compounds, which is correspond to A_2A_Rs expressions. However, most of the radioligands also showed a considerable degree of specific binding in the cerebral cortex and cerebrum, which is not observed with [11C]KF21213. Therefore, based on the uptake on the receptor poor cerebellum to receptor-rich striatum, [11C]KF21213 was found to be the most selective for A_2A_R, and followed by other representative A_2A_R PET tracers such as [11C]SCH442416 and [11C]TMSX (Bar-Yehuda et al., 2009; Mishina and Ishiwata, 2014).

## Positron Emission Tomography Imaging of A2A Receptors

### *In vivo* Positron Emission Tomography Imaging of Adenosine A_2A_ Receptors in Healthy Subjects

Based on the specific binding between the A_2A_ receptor ligand and the PET radioactive tracer, Ishiwata et al. directly visualized human brain adenosine A_2A_ receptors using [11C]TMSX PET (Leussis et al., 2008; Bar-Yehuda et al., 2009; Ishiwata et al., 2010). Theophylline stimulation confirmed the specific binding of [11C]TMSX to adenosine A_2A_ receptor (Ishiwata et al., 2005). Thus, the good reproducibility of [11C]TMSX PET in striatum was confirmed. The highest A_2A_ receptor density was observed in nucleus putamen in [11C]TMSX PET images, followed by caudate head and thalamus. And the relative low density of A_2A_ receptor was observed in cerebral cortex and frontal lobe. (Ishiwata et al., 2005; Leussis et al., 2008). Autopsy and non-human studies have found that [11C]TMSX PET shows great binding potential in the adenosine A_2A_ receptor-rich striatum, but [11C]TMSX binds more strongly in the human thalamus than in other mammals (Mishina and Ishiwata, 2014). Moreover, as the first non-xanthine A_2A_R PET tracer, [11C]SCH442416 showed highest binding in putamen and the lowest binding in cerebellar was observed in unaffected people (Brooks et al., 2010). The specific binding of [11C]SCH442416 was also calculated with cerebellum as the reference region to study the different binding potentials in the putamen by Ramlackhansingh et al., (2011).

With a good maximal striatal to cerebellar ratio in rodents but low in primates, [11C]SCH442416 was not suitable for the receptor occupancy quantification studies. Barret et al. used 18F to label a new compound (Barret et al., 2014), namely [18F]MNI-444, to solve this problem. Barret et al. reported the first whole-body biodistribution and dose estimates of [18F]MNI-444 in healthy controls. The high accumulation of [18F]MNI-444 was observed in the caudate and putamen, where the density of A_2A_ receptor is high, while the lower accumulation was discovered in the cortex and cerebellum. The distribution of MNI-444 in the brain is consistent with the known A_2A_ expressions reported by autoradiography and with previous observations in non-human primate brains. Therefore, [18F]MNI-444 holds the possibility to be a good PET tracer for imaging of A_2A_ receptors in the human brain (Barret et al., 2015). PET imaging with [18F]MNI-444 showed a rapid brain distribution, and the uptake pattern was consistent with known A_2A_R densities in the human brain. The favorable kinetic properties of [18F]MNI-444 may promote the PET imaging of A_2A_R in research related to neurodegenerative and psychiatric diseases.

What’s more, the clinical study of [11C]preladenant showed the individual organ and total-body administration of [11C]preladenant were comparable with other 11C-labeled tracers. As is known to all, the highest signal level of A_2A_Rs was observed in the basal ganglia, followed by cerebral cortex and thalamus. [11C]preladenant's regional distribution in healthy human brain is consistent with A_2A_R density. [11C]preladenant provides a feasible approach for imaging of adenosine A_2A_R in the brain. Therefore, A_2A_R density can be quantified using the cerebellum as a reference tissue model for the reference region. Further inhibition studies in the human brain may be needed to fully verify the existence of reference regions.

### Adenosine A_2A_ Receptor Positron Emission Tomography Imaging in Patients

As a novel and powerful imaging technology, PET and its clinical applications are expanding rapidly. Compared with other imaging technologies, PET possess unique characteristics such as high target specificity, quantitative ability, and high sensitivity, which can visualize and characterize receptor expressions during the development of disease. Several radio-tracers for A_2A_ receptors imaging have also been evaluated in human studies for the diagnosis of disease, including [11C]TMSX, [11C]SCH442416, [11C]preladenant, and [11C]KW6002, the characteristics and availability of these ligands are discussed below.

#### [11C]**TMSX**

Dynamic PET imaging using A_2A_R-specific [11C]TMSX was performed on progressive multiple sclerosis (SPMS) patients (Merskey, 1983), Parkinson's disease (PD) (Hirani et al., 2001), multiple sclerosis (Rissanen et al., 2013; Rissanen et al., 2015) and healthy controls (Merskey, 1983). The upregulated A_2A_R expression was observed in the brain of those patients, and these studies indicates that the [11C]TMSX dynamic PET can be used in the diagnosis of central nervous system (CNS) disorders. Studies using PET on the normal appearing of white matter (NAWM) in SPMS patients found that increased A_2A_R expression was correlated with decreased anisotropy score. This highlights the potential usefulness of TMSX-PET imaging in helping to detect normal appearing of white matter in diffuse lesions associated with progressive disease. Therefore, this method can make up for the deficiency of traditional imaging in diffuse change. Studies have shown an increase in [11C]TMSX binding in the putamen after anti-parkinsonian treatment. This finding may reflect compensatory changes in dopamine reduction in PD patients. Therefore, this may suggest that in PD patients, after anti-parkinsonian treatment, the increase of A2ARs in the putamina precedes the occurrence of dyskinesia. The application [11C]TMSX PET will help to further investigate the unknown mechanisms of side effects of anti-Parkinson drugs. Thus, [11C]TMSX-PET affords a novel method to diagnosis the pathology in CNS disorders (Li et al., 2019).

Moreover, Ishiwata reported that the highly uptake of [11C]TMSX in the myocardium suggested the specific binding of myocardial A_2A_R and [11C]TMSX can be used for myocardial PET imaging (Ishiwata et al., 2003a). The differences between the PET images generated by [11C]TMSX and the non-xanthine ligands is the signal-to-noise ratios, as TMSX holds higher affinity than other non-xanthine radiotracers (Li et al., 2019).

In addition, [11C]TMSX showed specific binding in peripheral tissues that was not detected by non-xanthine radiooligomer [11C]SCH442416, or [11C]Preladenant. [11C]TMSX can be used for brown adipose tissue (BAT) related A_2A_ imaging in addition to the central neural system and cardiovascular system. Lahesmaa et al. discovered that cold exposure stimulates the release of noepinephrine and significantly reduced the concentrations of available A_2A_R in BAT used for [11C]TMSX binding, demonstrating an increased endogenous adenosine release compared to baseline (Lahesmaa et al., 2019). [11C]TMSX binding with BAT decreases when BAT is exposed to cold, which indicates that endogenous adenosine and irradiated oligosaccharide competition receptors show high binding (Sousa and Diniz, 2017). Interestingly, the reduction of [11C]TMSX binding is related to increased perfusion in BAT, further indicating that endogenous adenosine release in BAT is accompanied by the increased oxidative metabolism. This implies that adenosine and A_2A_R are significant in the BAT activation induced by cold, which provides a new therapeutic direction for the fight against obesity and diabetes.

#### [11C]SCH442416

In order to avoid photoisomerization generated by xanthine analogues, Todde et al. labelled the first non-xanthine A_2A_ antagonist, [11C]SCH442416, whose kinetic behavior in rodents suggests that it may be used for *in vivo* imaging of the A_2A_ adenosine receptor in future (Todde et al., 2000). [11C]SCH442416, as an *in vivo* marker of A2A effectiveness, can selectively and reversibly bind to striatum A2A receptor with nanoscale affinity. PET imaging with [11C]SCH442416 was used to observe the expression of levodopa-induced dyskinesias (LIDs) in patients with Parkinson's disease (Ramlackhansingh et al., 2011). This implies that A2A antagonists may have value in levodopa-induced dyskinesias intervention while reducing levodopa dose. [11C]SCH442416 PET provides an efficient and robust approach for *in vivo* studies of the effectiveness of A2A. [11C]SCH442416 PET also can be used to determine the dose occupation of other A2A antagonists. In addition, [18F]FESCH and [18F]FPSCH are prepared as the analogs of SCH442416 (Khanapur et al., 2014; Khanapur et al., 2017).

#### [11C]**Preladenant**

[11C]SCH442416 and [11C]TMSX are the most favorable tracers for imaging A_2A_Rs in brain. However, low target-to-nontarget ratios, high nonspeciﬁc binding and low binding potentials are the disadvantages of these tracers. Thus, the newly improved radioactive ligand [11C]preladenant was developed for imaging A_2A_Rs in the living brain, including human brain, rat brain and monkey brain (Sakata et al., 2017; Zhou et al., 2017a; Zhou et al., 2017b; Zhou et al., 2017c). It is a non-xanthine heterocyclic compound with high selectivity, sufficient affinity for image receptors without affecting the quantification of receptors, and this compound also showed good pharmacokinetic properties (Zhou et al., 2014). With superior target-to-nontarget ratios and excellent pharmacokinetic properties, this tracer was advanced into human studies. Recently, studies have shown that [11C]preladenant is applied to healthy human brains in a manner consistent with A_2A_R density. Thus, it indicated that [11C]preladenant is suitable for imaging of A_2A_Rs in the living brain (Sakata et al., 2017). In addition, compared with other ARs, [11C]preladenant showed high affinity and significant selectivity for A_2A_R (Neustadt et al., 2007; Zhou et al., 2014). Recently, Ishibashi et al. reported [11C]preladenant PET can be used to calculate the occupancy rate of Istradefylline to A_2A_R (Ishibashi et al., 2018). These results demonstrated that [11C]preladenant is a suitable tracer to evaluate A2A receptor occupancy and quantify striatal A2A receptor density by A2A receptor-targeting molecules (Sakata et al., 2017; Zhou et al., 2017a; Zhou et al., 2017c).Thus, [11C]preladenant PET is suitable for non-invasive A_2A_R quantification and evaluation of A_2A_R occupation in A_2A_R abundant regions in living brain.

#### [11C]KW6002

In healthy rat, although [11C]KW-6002 shows some potential as a PET ligand, it also showed low cerebral cortex and cerebellar retention, and it may proved to be insufﬁciently selective to be a useful *in vivo* radio-tracer, at least in rodents; however, it also binds to the outer fissure region, so its potential as a PET tracer needs further studies (Hirani et al., 2001). In primate and rodent models, KW6002 offers symptomatic relief of Parkinson's motor deficits without causing or exacerbating previous motor deficits. A human study of KW6002 in advanced PD patients with levodopa-related motor complications yielded good results in the remission of motor symptoms without the side effects of exercise (Bar-Yehuda et al., 2009). The uptake of [11C]KW-6002 in the brain was characterized by a blood volume term in the two-compartment model and a 50% effective dose (ED50) of cold KWL-6002 in the striatum at 0.5 mg (Bar-Yehuda et al., 2009). In humans, [11C]KW-6002 blocks were observed in all brain regions studied, which may be caused by non-specific binding to A_1_R and A_2B_R. In addition, may be due to the non-specific binding, [11C]KW-6002 has not been further studied.

## Summary

Extracellular adenosine is an important regulatory molecule that interacts with four ARs: A1R, A2AR, A2BR and A3R through intracellular adenosine regulating the physiological function of the cell. Changes in function and expression in neurological disorder (Parkinson's disease, Alzheimer's disorder, epilepsy), inflammation, cardiovascular disease, autoimmune diseases, and cancer were studied. A series of PET tracers for ARs were developed. Of all the tracers listed, [11C]TMSX is the oldest ligand and has been widely evaluated in several mammal populations. Since the 1990s, several radioligands have been produced for brain A2ARs PET imaging. These ligands suitable for studying humans include [11C]TMSX, [11C]SCH442416, [11C]preladenant, [11C]KW-6002, [18F]MNI-444. It seems that the xanthine scaffold may provide efficient binding specificity for the A2AR subtype. However, photoisomerization should be taken into consideration when developing xanthine-type adenosine A2A receptor-selective ligands.

Although adenosine can also be tested by *in situ* hybridization and immunochemistry probes in recent years, PET imaging of A_2A_R can further be used to capture changes in A_2A_Rs distribution and density as the disease progresses, as well as to monitor treatment responses to these changes. In addition, PET can also determine the A_2A_R occupancy in the brain can be measured by PET, and hence providing a useful method for drug discovery (Tavares et al., 2013). The PET radio-tracers provided valuable information for the diagnosis and treatment of diseases associated with altered ARs expression, following of the summary picture.

Molecular imaging plays a crucial role in improving accuracy by quantifying, characterizing and visualizing biological processes at the molecular and cellular levels in living body, which provides an achievable basis for precision medicine. Therefore, how to realize the personalized diagnosis and treatment of A2A-related diseases with PET imaging technology will become an important research direction in the future. In addition, the application of PET molecular imaging technology in assessing A2A disease risk and understanding disease mechanisms would also make a significant contribution to the medical profession.

## Author Contributions

MS and YZ prepared the manuscript, review and editing by XW and FL.

## Funding

This work is supported by Sichuan Science and Technology Program (No. 2017JY0324).

## Conflict of Interest

The authors declare that the research was conducted in the absence of any commercial or financial relationships that could be construed as a potential conflict of interest.
